# Synthesis, structure and Hirshfeld surface analysis of 1,3-bis­[(1-octyl-1*H*-1,2,3-triazol-4-yl)meth­yl]-1*H*-benzo[*d*]imidazol-2(3*H*)-one

**DOI:** 10.1107/S2056989023009891

**Published:** 2023-11-21

**Authors:** Mustapha Zouhair, Lhoussaine El Ghayati, Hanae El Monfalouti, Hicham Abchihi, Tuncer Hökelek, Mazzah Ahmed, Joel T. Mague, Nada Kheira Sebbar

**Affiliations:** a Laboratory of Heterocyclic Organic Chemistry, Medicines Science Research Center, Pharmacochemistry Competence Center, Mohammed V University in Rabat, Faculté des Sciences, Av. Ibn Battouta, BP 1014, Rabat, Morocco; b Laboratory of Plant Chemistry, Organic and Bioorganic Synthesis, Faculty of Sciences, Mohammed V University in Rabat, 4 Avenue Ibn Battouta, BP 1014 RP, Morocco; c Department of Physics, Hacettepe University, 06800 Beytepe, Ankara, Türkiye; d Science and Technology of Lille USR 3290, Villeneuve d’ascq cedex, France; e Department of Chemistry, Tulane University, New Orleans, LA 70118, USA; f Laboratory of Organic and Physical Chemistry, Applied Bioorganic Chemistry Team, Faculty of Sciences, Ibn Zohr University, Agadir, Morocco; University of Aberdeen, United Kingdom

**Keywords:** crystal structure, benzimidazolone, triazole, C—H⋯π(ring) inter­action, hydrogen bond

## Abstract

The title mol­ecule, C_29_H_44_N_8_O, adopts a conformation resembling a two-bladed fan with the octyl chains largely in fully extended conformations. In the crystal, C—H⋯O hydrogen bonds form chains of mol­ecules extending along the *b*-axis direction, which are linked by weak C—H⋯N hydrogen bonds and C—H⋯π inter­actions to generate a three-dimensional network. A Hirshfeld surface analysis of the crystal structure indicates that the most important contributions for the crystal packing are from H⋯H (68.3%), H⋯N/N⋯H (15.7%) and H⋯C/C⋯H (10.4%) inter­actions.

## Abstract 2

The title mol­ecule adopts a conformation resembling a two-bladed fan with the octyl chains in fully extended conformations. In the crystal, the mol­ecules are linked by C—H⋯O and C—H⋯N hydrogen bonds and C—H⋯π inter­actions.

## 1. Chemical context

Benzimidazolone derivatives display diverse pharmacological and biological properties including anti­viral (Ferro *et al.*, 2017 ▸), anti­bacterial (Saber *et al.*, 2020 ▸; Menteşe *et al.*, 2021 ▸), anti­cancer (Guillon *et al.*, 2022 ▸), anti-Alzheimer’s (Mo *et al.*, 2020 ▸), anti­fungal (Ibrahim *et al.*, 2021 ▸), and anti­oxidant (Ibrahim *et al.*, 2021 ▸) activities. In our ongoing research in this area, we are synthesizing compounds that combine the 1,2,3-triazole motif with benzimidazol-2-one derivatives. In this report, we present the synthesis and structure of the title compound, C_29_H_44_N_8_O, which was obtained using click chemistry, specifically the copper-catalysed azide–alkyne cyclo­addition (CuAAC) method. Additionally, we describe the Hirshfeld surface analysis and calculations on crystal voids and inter­molecular inter­action energies and energy frameworks. 
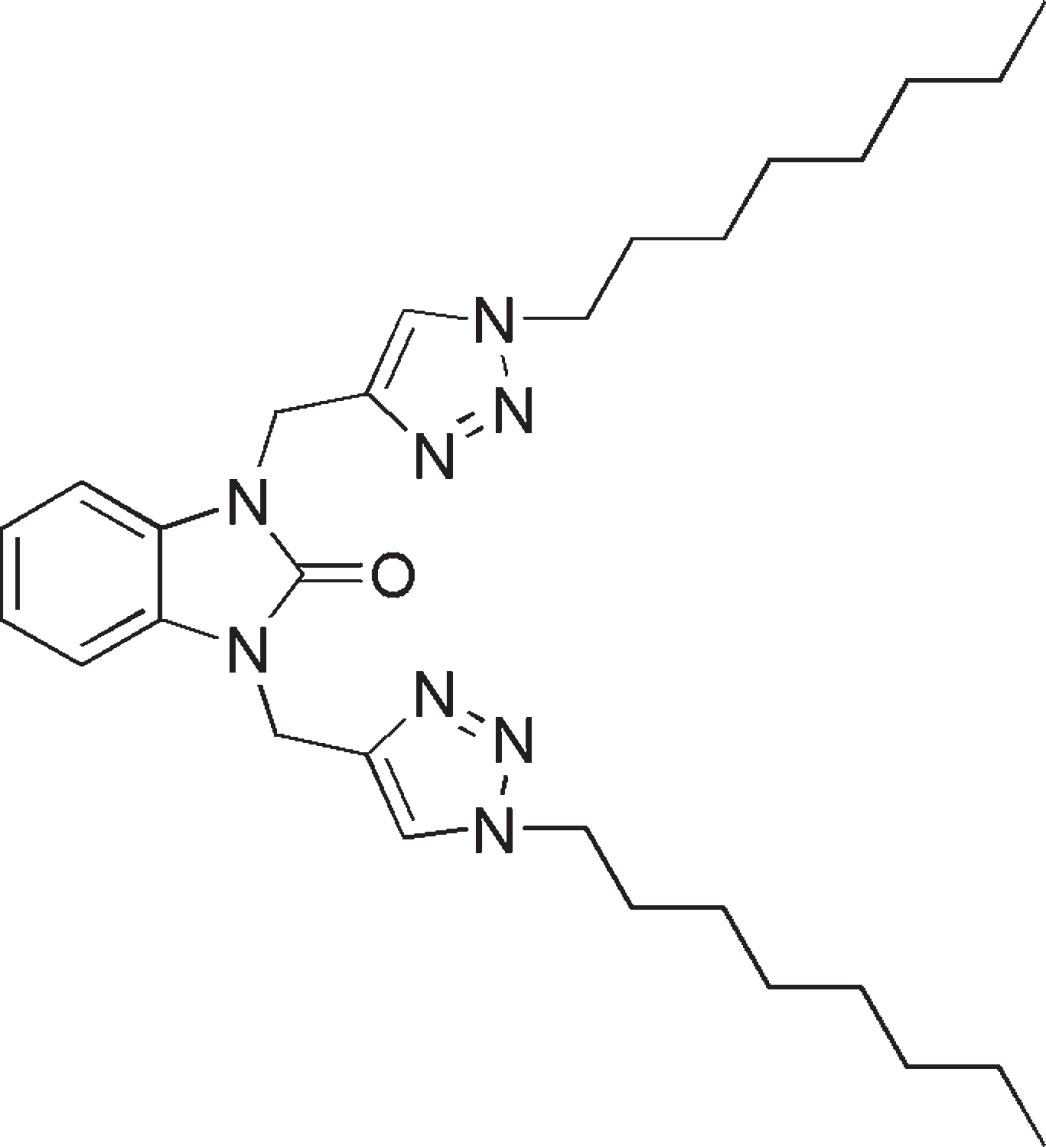


## 2. Structural commentary

**Figure 1:**
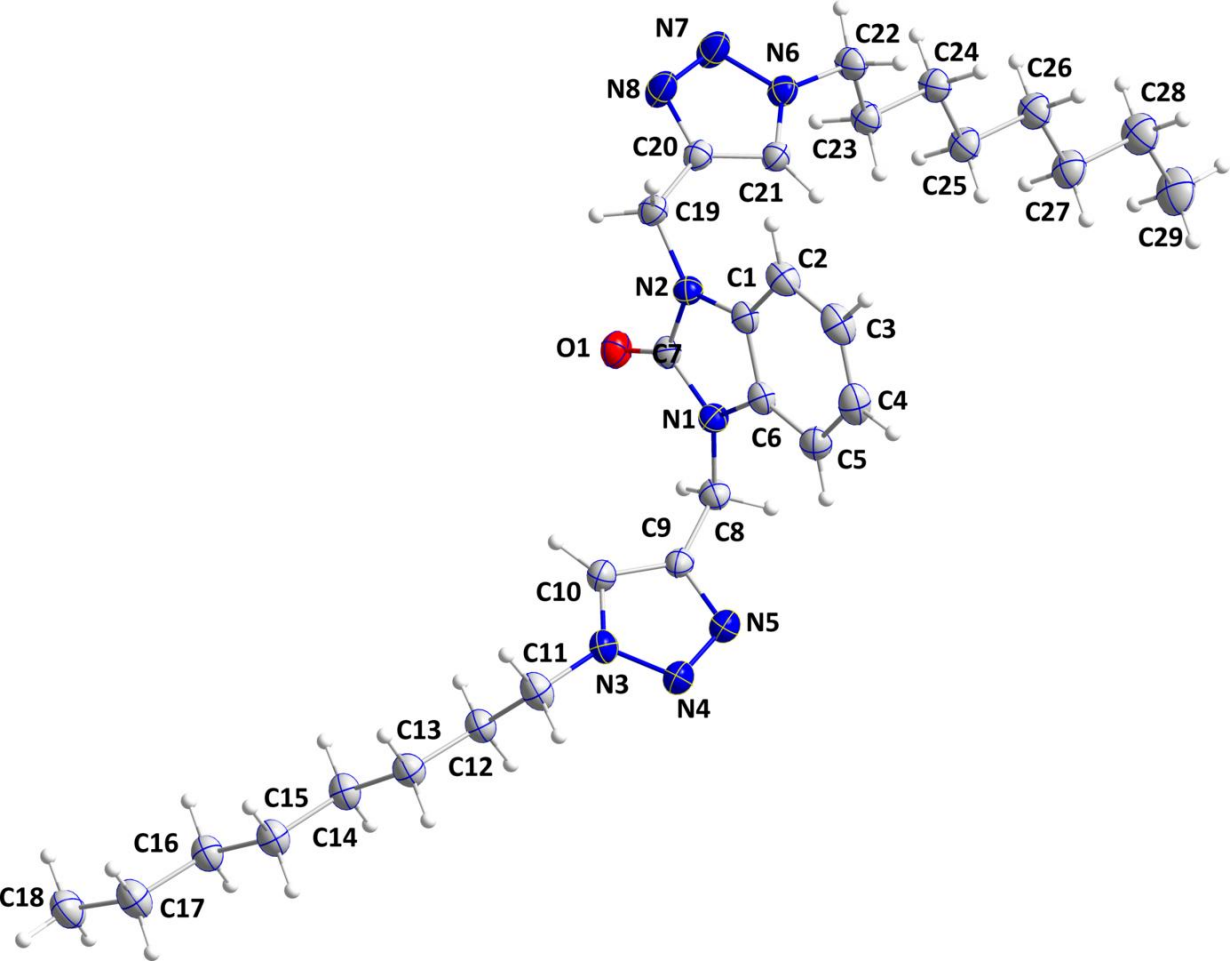
The title mol­ecule with 50% probability ellipsoids.

The title mol­ecule adopts a conformation similar to a two-bladed fan in which the octyltriazolylmethyl substituents extend in opposite directions from the benzimidazolone core (Fig. 1 ▸). The C1–C7/N1/N2 benzimidazole moiety is planar to within 0.0155 (13) Å (r.m.s. deviation = 0.007 Å) and the mean planes of the C9/C10/N3–N5 and C20/C21/N6–N8 rings are inclined to the above plane by 75.72 (6) and 83.07 (6)°, respectively: the dihedral angle between the pendant heterocyclic rings is 7.37 (11)°. Both octyl chains have a modest kink near the triazole ring as indicated by the C11—C12—C13—C14 and C22—C23—C24—C25 torsion angles of 169.67 (17) and 168.59 (16)°, respectively. Otherwise, both are in fully extended conformations with the remaining torsion angles differing by no more than about 4° from ±180° (Fig. 1 ▸).

## 3. Supra­molecular features

**Figure 2:**
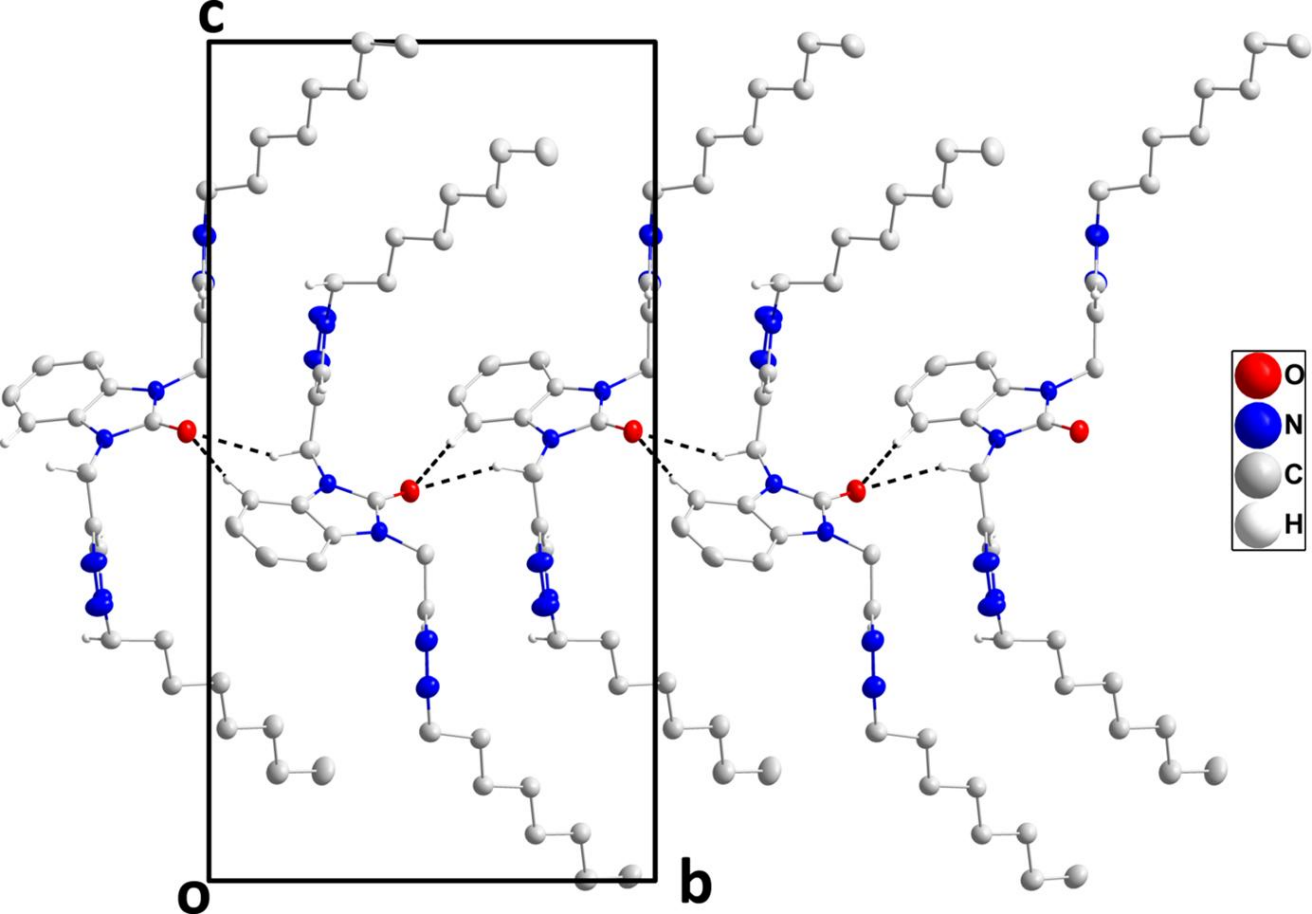
A portion of one chain of mol­ecules viewed along the *a*-axis direction with C—H⋯O hydrogen bonds depicted by dashed lines and non-inter­acting hydrogen atoms omitted for clarity.

**Figure 3:**
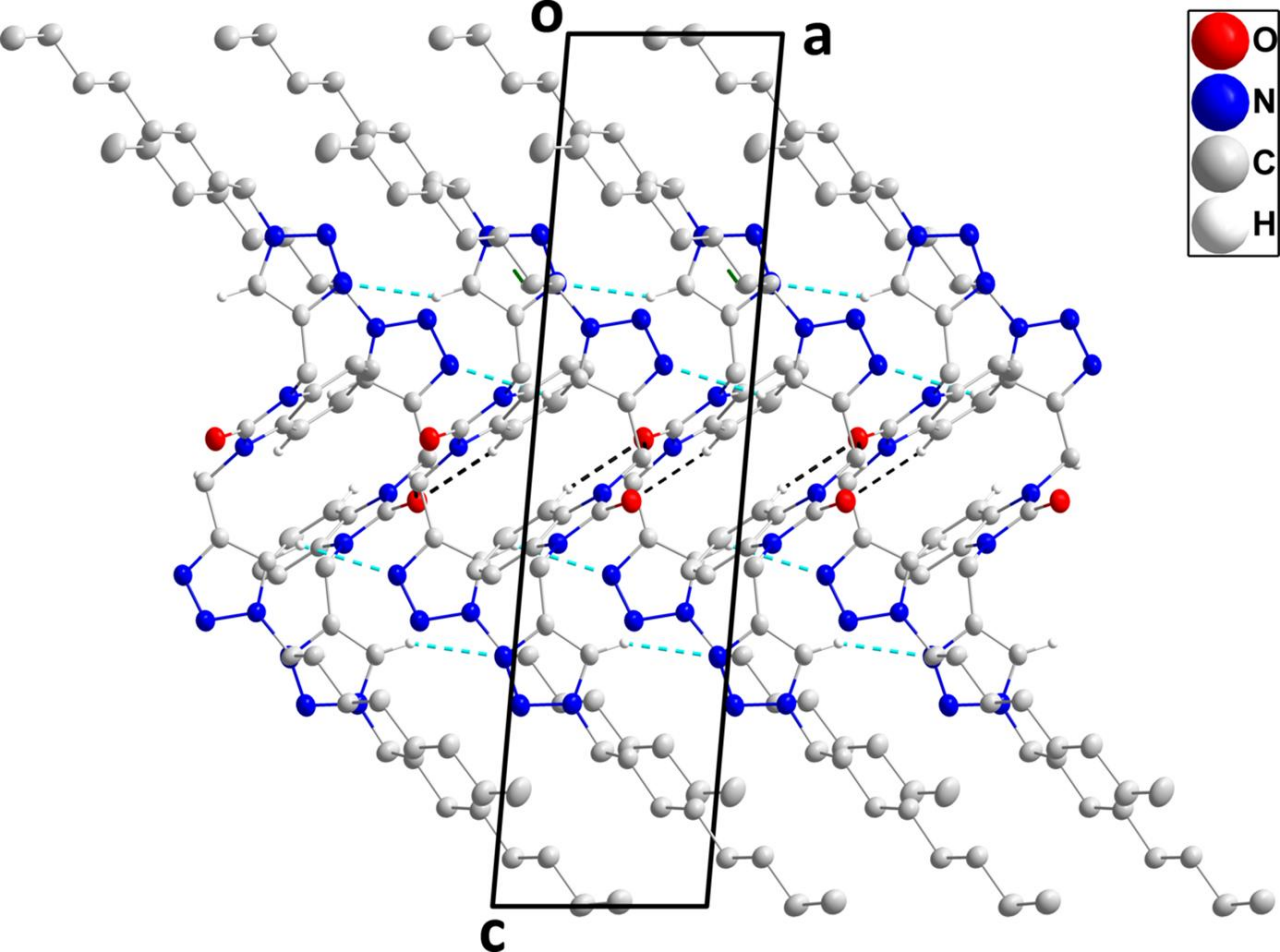
Packing viewed along the *b*-axis direction with C—H⋯O and C—H⋯N hydrogen bonds depicted, respectively, by black and light-blue dashed lines. The C—H⋯π(ring) inter­actions are depicted by dark-green dashed lines and non-inter­acting hydrogen atoms omitted for clarity.

In the crystal, C2—H2⋯O1 and C19—H19*A*⋯O1 hydrogen bonds (Table 1 ▸) form chains of mol­ecules extending along the *b*-axis direction (Fig. 2 ▸). The chains are cross-linked by weak C10—H10⋯N5 and C21—H21⋯N8 hydrogen bonds and by C22—H22*A*⋯*Cg*2 inter­actions (Table 1 ▸) into a three-dimensional network (Fig. 3 ▸).

## 4. Hirshfeld surface analysis and computational chemistry

**Figure 4:**
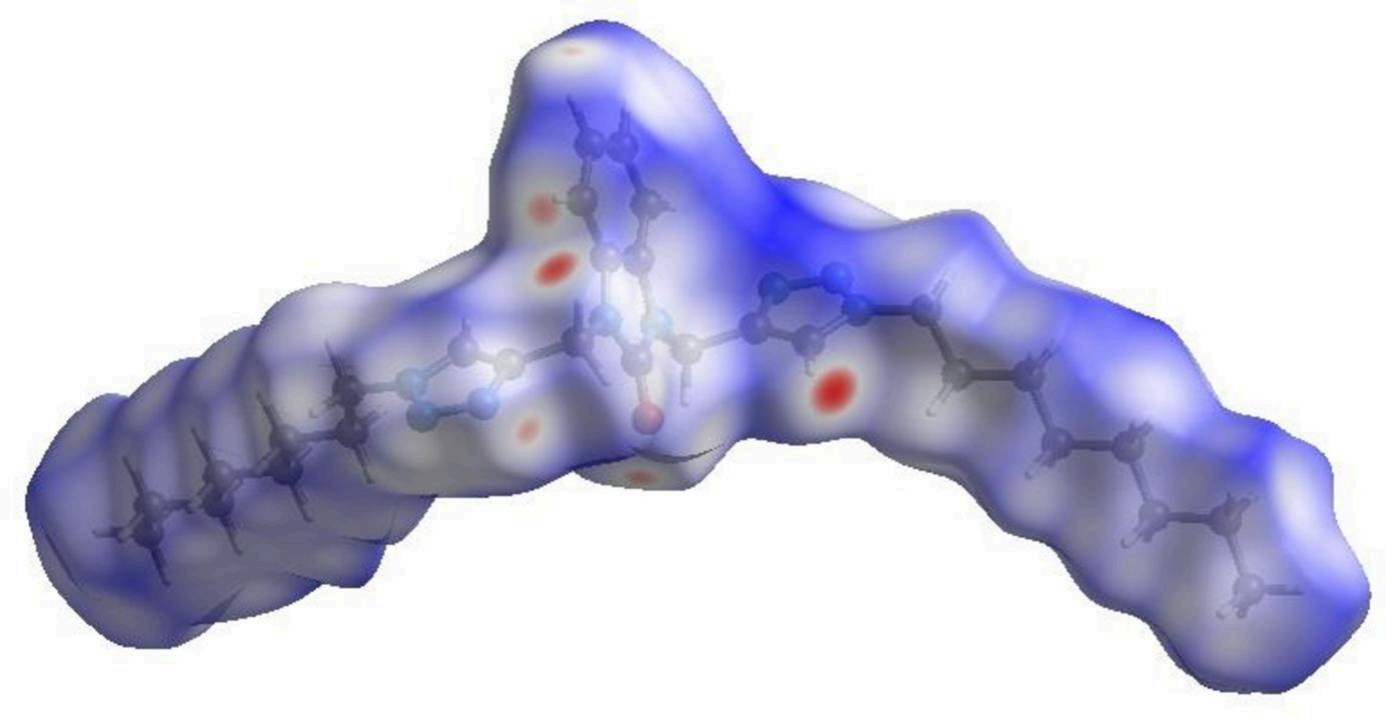
View of the three-dimensional Hirshfeld surface of the title compound plotted over *d*_norm_ in the range −0.24 to 1.57 a.u.

**Figure 5:**
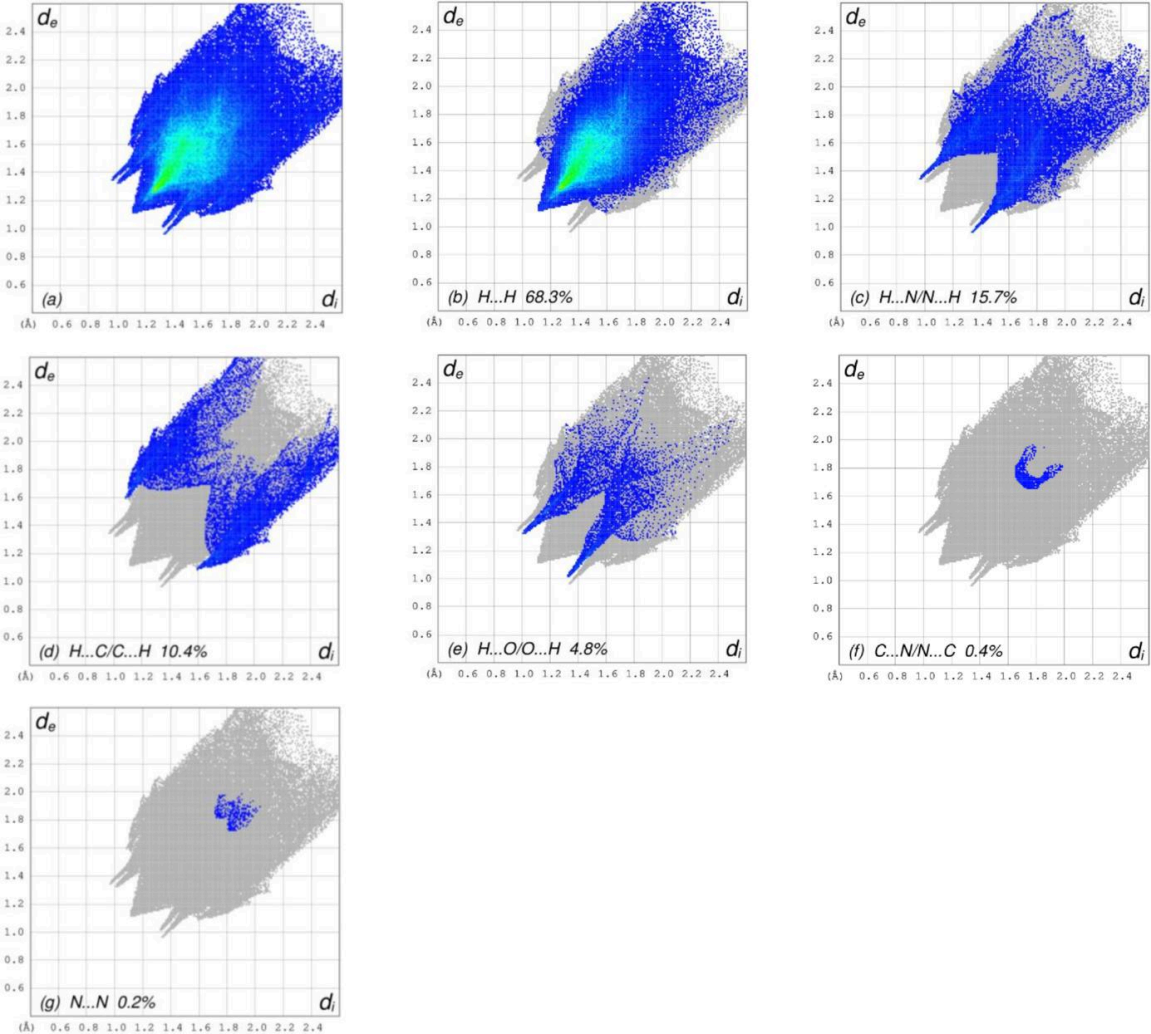
The full two-dimensional fingerprint plots for the title compound, showing (*a*) all inter­actions, and delineated into (*b*) H⋯H, (*c*) H⋯N/N⋯H, (*d*) H⋯C/C⋯H, (*e*) H⋯O/O⋯H, (*f*) C⋯N/N⋯C and (*g*) N⋯N inter­actions. The *d*_i_ and *d*_e_ values are the closest inter­nal and external distances (in Å) from given points on the Hirshfeld surface.

In order to further visualize the inter­molecular inter­actions in the crystal of the title compound, a Hirshfeld surface (HS) analysis was carried out by using *Crystal Explorer 17.5* (Turner *et al.*, 2017 ▸), as shown in Fig. 4 ▸. The overall two-dimensional fingerprint plot, Fig. 5 ▸ *a*, and those delineated into H⋯H, H⋯N/N⋯H, H⋯C/C⋯H, H⋯O/O⋯H, C⋯N/N⋯C and N⋯N (McKinnon *et al.*, 2007 ▸) are illustrated in Fig. 5 ▸ *b*–*g* respectively, together with their relative contributions to the Hirshfeld surface. The most important inter­action is H⋯H, contributing 68.3% to the overall crystal packing, which is reflected in Fig. 7*b* as widely scattered points of high density, due to the large hydrogen content of the mol­ecule, with the tip at *d*_e_ = *d*_i_ = 1.12 Å. The pair of characteristic wings in the fingerprint plot delineated into H⋯N/N⋯H contacts (15.7% contribution to the HS; Fig. 5 ▸ *c*) is viewed as pair of spikes with the tips at *d*_e_ + *d*_i_ = 2.30 Å. In the presence of C—H⋯π inter­actions, the H⋯C/C⋯H contacts, contributing 10.4% to the overall crystal packing, are reflected in Fig. 5 ▸ *d* with the tips at *d*_e_ + *d*_i_ = 2.69 Å. The pair of characteristic wings in the fingerprint plot delineated into H⋯O/O⋯H contacts (4.8% contribution to the HS; Fig. 5 ▸ *e*) is viewed as pair of spikes with the tips at *d*_e_ + *d*_i_ = 2.32 Å. Finally, the C⋯N/N⋯C (Fig. 5 ▸ *f*) and N⋯N (Fig. 5 ▸ *g*) contacts, with 0.4% and 0.2% contributions, respectively, to the HS, have very low distributions of points.

A void analysis was performed by summing the electron densities of the spherically symmetric atoms contained in the asymmetric unit (Turner *et al.*, 2011 ▸). The void surface is defined as an isosurface of the procrystal electron density and is calculated for the whole unit cell where the void surface meets the boundary of the unit cell and capping faces are generated to create an enclosed volume. The volume of the crystal voids (supplementary Fig. S1) and the percentage of free space in the unit cell are calculated to be 198.6 and 13.4 Å^3^, respectively.

The inter­molecular inter­action energies were calculated using the CE–B3LYP/6–31G(d,p) energy model available in *Crystal Explorer 17.5* (Turner *et al.*, 2017 ▸). The total inter­molecular energy (*E*_tot_) is the sum of electrostatic (*E*_ele_), polarization (*E*_pol_), dispersion (*E*_dis_) and exchange–repulsion (*E*_rep_) energies (Turner *et al.*, 2015 ▸) with scale factors of 1.057, 0.740, 0.871 and 0.618, respectively (Mackenzie *et al.*, 2017 ▸). Energy frameworks were constructed for *E*_ele_ (red cylinders), *E*_dis_ (green cylinders) and *E*_tot_ (blue cylinders) (supplementary Fig. 2*a* and 2*b*). These data indicate that dispersion energy is the most important contributor to the cohesion of the crystal structure of the title compound. The theoretical optimization of the title structure in the gas phase was conducted by density functional theory (DFT), using the standard B3LYP functional and 6-311 G(d,p) basis-set calculations (Becke, 1993 ▸). The energy band gap [Δ*E* = *E*_LUMO_ – *E*_HOMO_] of the mol­ecule is 5.04 eV, and the frontier mol­ecular orbitals, *E*_HOMO_ and *E*_LUMO_ have relative energies of −5.72 and 0.68 eV, respectively (supplementary Tables 1 and 2 and supplementary Fig. S3).

## 5. Database survey

A survey of the Cambridge Structural Database (CSD, Version 5.42, last update February 2023; Groom *et al.*, 2016 ▸) for structures similar to the title mol­ecule gave hits for compound **I** with *R*_1_ = H, *R*_2_ = –CH_2_C_6_H_5_ and *R*_3_ = –OCH_3_ (CSD refcode HIJXAC; El Bakri *et al.*, 2018 ▸), **II** with *R*_1_ = –C_6_H_9_, *R*_2_ = –C_6_H_5_ and *R*_3_ = –H (PAZFOO; Adardour *et al.*, 2017 ▸), **III** with *R*_1_ = –C(CH_3_)=CH_2_, *R*_2_ = –C_10_H_22_ and *R*_3_ = –H (ETAJOB; Saber *et al.*, 2021 ▸) and **IV** with *R*_1_ = –CH_2_C_6_H_5_, *R*_2_ = –C_12_H_26_ and *R*_3_ = –H (ETAKAO; Saber *et al.*, 2021 ▸). 
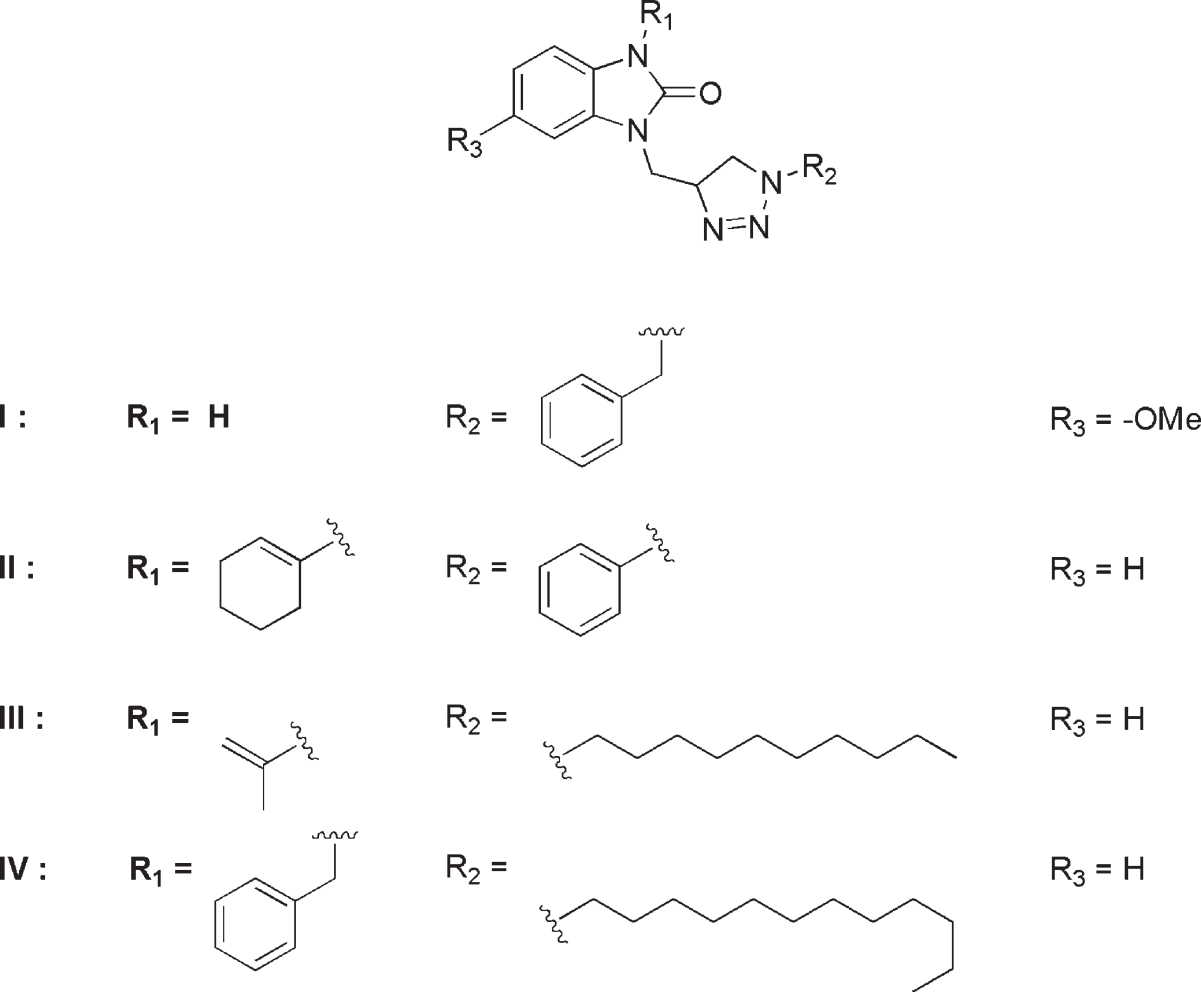


## 6. Synthesis and crystallization

To a solution of 1.64 mmol of 1,3-di(prop-2-yn­yl)-1*H*-benzimidazol-2-one and 2 mmol of 1-azido­octane in 15 ml of ethanol were added 1.15 mmol of CuSO_4_ and 2.62 mmol of sodium ascorbate dissolved in 10 ml of distilled water. The reaction mixture was stirred for 10 h at room temperature and monitored by TLC. After filtration and concentration of the solution under reduced pressure, the residue obtained was chromatographed on a silica gel column using ethyl acetate/hexane (3/1) as eluent. Colourless plates of the title compound in 73% yield were recrystallized from ethanol solution.

## 7. Refinement

Crystal data, data collection and structure refinement details are summarized in Table 2 ▸. H atoms were positioned with idealized geometry (C—H = 0.95–0.99 Å) and refined isotropically with *U*_iso_(H) = 1.2–1.5*U*_eq_(C) using a riding model.

## Supplementary Material

Crystal structure: contains datablock(s) global, I. DOI: 10.1107/S2056989023009891/hb8081sup1.cif

Structure factors: contains datablock(s) I. DOI: 10.1107/S2056989023009891/hb8081Isup2.hkl

Supporting information file. DOI: 10.1107/S2056989023009891/hb8081Isup3.cdx

Supporting information file. DOI: 10.1107/S2056989023009891/hb8081Isup5.cml

Supplementary figures: void volumes, energy frameworks and HOMO and LUMO. DOI: 10.1107/S2056989023009891/hb8081sup4.docx

CCDC reference: 2307930

Additional supporting information:  crystallographic information; 3D view; checkCIF report

## Figures and Tables

**Table 1:** Hydrogen-bond geometry (Å, °) *Cg*2 is the centroid of the N3–N5/C9/C10 ring.

*D*—H⋯*A*	*D*—H	H⋯*A*	*D*⋯*A*	*D*—H⋯*A*
C2—H2⋯O1^i^	0.95	2.59	3.502 (2)	162
C10—H10⋯N5^ii^	0.95	2.44	3.317 (2)	153
C19—H19*A*⋯O1^i^	0.99	2.43	3.334 (2)	152
C21—H21⋯N8^iii^	0.95	2.62	3.372 (2)	137
C22—H22*A*⋯*Cg*2^iv^	0.99	2.89	3.664 (2)	135

Symmetry codes: (i) 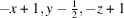 ; (ii) 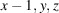 ; (iii) 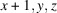 ; (iv) 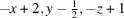 .

**Table 2:** Experimental details

Crystal data	
Chemical formula	C_29_H_44_N_8_O
*M*_r_	520.72
Crystal system, space group	Monoclinic, *P*2_1_
Temperature (K)	150
*a*, *b*, *c* (Å)	5.5229 (2), 11.9579 (5), 22.5767 (9)
β (°)	94.962 (1)
*V* (Å^3^)	1485.43 (10)
*Z*	2
Radiation type	Mo *K*α
μ (mm^−1^)	0.07
Crystal size (mm)	0.43 × 0.24 × 0.04

Data collection	
Diffractometer	Bruker D8 QUEST PHOTON 3 diffractometer
Absorption correction	Numerical (*SADABS*; Krause *et al.*, 2015 ▸)
*T*_min_, *T*_max_	0.97, 1.00
No. of measured, independent and observed [*I* > 2σ(*I*)] reflections	44175, 7403, 6607
*R*_int_	0.040
(sin θ/λ)_max_ (Å^−1^)	0.668

Refinement	
*R*[*F* ^2^ > 2σ(*F* ^2^)], *wR*(*F* ^2^), *S*	0.036, 0.090, 1.05
No. of reflections	7403
No. of parameters	346
No. of restraints	1
H-atom treatment	H-atom parameters constrained
Δρ_max_, Δρ_min_ (e Å^−3^)	0.21, −0.15
Absolute structure	Refined as an inversion twin
Absolute structure parameter	0.2 (13)

Computer programs: *APEX3* and *SAINT* (Bruker, 2020 ▸), *SHELXT* (Sheldrick, 2015*a* ▸), *SHELXL2018/1* (Sheldrick, 2015*b* ▸), *DIAMOND* (Brandenburg & Putz, 2012 ▸) and *SHELXTL* (Sheldrick, 2008 ▸).

## supplementary crystallographic information

### Crystal data

**Table:**

C_29_H_44_N_8_O	*F*(000) = 564
*M**_r_* = 520.72	*D*_x_ = 1.164 Mg m^−^^3^
Monoclinic, *P*2_1_	Mo *K*α radiation, λ = 0.71073 Å
*a* = 5.5229 (2) Å	Cell parameters from 9942 reflections
*b* = 11.9579 (5) Å	θ = 2.5–28.3°
*c* = 22.5767 (9) Å	µ = 0.07 mm^−^^1^
β = 94.962 (1)°	*T* = 150 K
*V* = 1485.43 (10) Å^3^	Plate, colourless
*Z* = 2	0.43 × 0.24 × 0.04 mm

### Data collection

**Table:**

Bruker D8 QUEST PHOTON 3 diffractometer	7403 independent reflections
Radiation source: fine-focus sealed tube	6607 reflections with *I* > 2σ(*I*)
Graphite monochromator	*R*_int_ = 0.040
Detector resolution: 7.3910 pixels mm^-1^	θ_max_ = 28.3°, θ_min_ = 1.8°
φ and ω scans	*h* = −7→7
Absorption correction: numerical (*SADABS*; Krause *et al.*, 2015)	*k* = −15→15
*T*_min_ = 0.97, *T*_max_ = 1.00	*l* = −30→30
44175 measured reflections	

### Refinement

**Table:**

Refinement on *F*^2^	Secondary atom site location: difference Fourier map
Least-squares matrix: full	Hydrogen site location: inferred from neighbouring sites
*R*[*F*^2^ > 2σ(*F*^2^)] = 0.036	H-atom parameters constrained
*wR*(*F*^2^) = 0.090	*w* = 1/[σ^2^(*F*_o_^2^) + (0.0469*P*)^2^ + 0.1166*P*] where *P* = (*F*_o_^2^ + 2*F*_c_^2^)/3
*S* = 1.05	(Δ/σ)_max_ < 0.001
7403 reflections	Δρ_max_ = 0.21 e Å^−^^3^
346 parameters	Δρ_min_ = −0.15 e Å^−^^3^
1 restraint	Absolute structure: Refined as an inversion twin
Primary atom site location: dual	Absolute structure parameter: 0.2 (13)

### Special details

**Table:**

Experimental. The diffraction data were obtained from 7 sets of frames, each of width 0.5° in ω or φ, collected with scan parameters determined by the "strategy" routine in *APEX3*. The scan time was 25 sec/frame.
Geometry. All esds (except the esd in the dihedral angle between two l.s. planes) are estimated using the full covariance matrix. The cell esds are taken into account individually in the estimation of esds in distances, angles and torsion angles; correlations between esds in cell parameters are only used when they are defined by crystal symmetry. An approximate (isotropic) treatment of cell esds is used for estimating esds involving l.s. planes.
Refinement. Refinement of F^2^ against ALL reflections. The weighted R-factor wR and goodness of fit S are based on F^2^, conventional R-factors R are based on F, with F set to zero for negative F^2^. The threshold expression of F^2^ > 2sigma(F^2^) is used only for calculating R-factors(gt) etc. and is not relevant to the choice of reflections for refinement. R-factors based on F^2^ are statistically about twice as large as those based on F, and R- factors based on ALL data will be even larger. H-atoms attached to carbon were placed in calculated positions (C—H = 0.95 - 0.99 Å). All were included as riding contributions with isotropic displacement parameters 1.2 - 1.5 times those of the attached atoms. Refined as a 2-component inversion twin.

### Fractional atomic coordinates and isotropic or equivalent isotropic displacement parameters (Å^2^)

**Table:**

	*x*	*y*	*z*	*U*_iso_*/*U*_eq_	
O1	0.5165 (2)	0.45034 (11)	0.46448 (6)	0.0329 (3)	
N1	0.8422 (3)	0.38162 (12)	0.41592 (6)	0.0266 (3)	
N2	0.6546 (3)	0.26610 (12)	0.47315 (6)	0.0256 (3)	
N3	0.7105 (3)	0.48912 (14)	0.23204 (7)	0.0308 (3)	
N4	0.9528 (3)	0.49012 (15)	0.23032 (7)	0.0357 (4)	
N5	1.0483 (3)	0.48764 (14)	0.28579 (7)	0.0343 (4)	
N6	0.7789 (3)	0.26119 (13)	0.66306 (6)	0.0276 (3)	
N7	0.5441 (3)	0.25415 (17)	0.67279 (7)	0.0402 (4)	
N8	0.4205 (3)	0.24210 (16)	0.62051 (7)	0.0377 (4)	
C1	0.8325 (3)	0.20418 (14)	0.44791 (8)	0.0261 (4)	
C2	0.8978 (4)	0.09248 (15)	0.45373 (9)	0.0316 (4)	
H2	0.816702	0.042829	0.478248	0.038*	
C3	1.0882 (4)	0.05678 (16)	0.42184 (9)	0.0358 (4)	
H3	1.138081	−0.019205	0.424677	0.043*	
C4	1.2070 (4)	0.12896 (17)	0.38608 (9)	0.0363 (4)	
H4	1.337097	0.101465	0.365294	0.044*	
C5	1.1398 (3)	0.24138 (16)	0.37988 (8)	0.0308 (4)	
H5	1.219570	0.290810	0.354969	0.037*	
C6	0.9523 (3)	0.27692 (14)	0.41167 (7)	0.0256 (3)	
C7	0.6557 (3)	0.37503 (15)	0.45279 (7)	0.0255 (3)	
C8	0.9182 (3)	0.48505 (15)	0.38886 (8)	0.0286 (4)	
H8A	1.094756	0.495617	0.398973	0.034*	
H8B	0.832786	0.548675	0.405854	0.034*	
C9	0.8673 (3)	0.48614 (15)	0.32269 (8)	0.0269 (3)	
C10	0.6497 (3)	0.48710 (16)	0.28838 (8)	0.0304 (4)	
H10	0.490916	0.486484	0.301568	0.037*	
C11	0.5501 (4)	0.49558 (17)	0.17728 (9)	0.0363 (4)	
H11A	0.424118	0.436762	0.177800	0.044*	
H11B	0.645578	0.481045	0.142950	0.044*	
C12	0.4278 (3)	0.60839 (16)	0.16948 (8)	0.0316 (4)	
H12A	0.553863	0.666846	0.167562	0.038*	
H12B	0.337533	0.624078	0.204577	0.038*	
C13	0.2529 (4)	0.61468 (16)	0.11355 (8)	0.0325 (4)	
H13A	0.347180	0.612977	0.078210	0.039*	
H13B	0.145729	0.548191	0.111835	0.039*	
C14	0.0970 (4)	0.71974 (16)	0.11140 (9)	0.0325 (4)	
H14A	0.205307	0.785813	0.114617	0.039*	
H14B	0.000482	0.720037	0.146372	0.039*	
C15	−0.0758 (4)	0.73133 (16)	0.05545 (9)	0.0340 (4)	
H15A	0.020707	0.736967	0.020592	0.041*	
H15B	−0.176824	0.663028	0.050563	0.041*	
C16	−0.2417 (4)	0.83263 (17)	0.05645 (9)	0.0346 (4)	
H16A	−0.140055	0.900797	0.060959	0.042*	
H16B	−0.335843	0.827341	0.091726	0.042*	
C17	−0.4187 (4)	0.84527 (18)	0.00121 (9)	0.0418 (5)	
H17A	−0.325170	0.855305	−0.033890	0.050*	
H17B	−0.514793	0.775659	−0.004678	0.050*	
C18	−0.5909 (4)	0.9431 (2)	0.00497 (10)	0.0454 (5)	
H18A	−0.697235	0.948212	−0.031997	0.068*	
H18B	−0.497132	1.012380	0.010853	0.068*	
H18C	−0.689828	0.931993	0.038498	0.068*	
C19	0.4857 (3)	0.22611 (15)	0.51395 (8)	0.0288 (4)	
H19A	0.453376	0.145736	0.506273	0.035*	
H19B	0.329889	0.266739	0.506328	0.035*	
C20	0.5775 (3)	0.24109 (14)	0.57782 (8)	0.0257 (3)	
C21	0.8082 (3)	0.25252 (15)	0.60465 (7)	0.0259 (3)	
H21	0.956179	0.254009	0.586136	0.031*	
C22	0.9617 (3)	0.28057 (16)	0.71286 (8)	0.0312 (4)	
H22A	1.096178	0.226424	0.710619	0.037*	
H22B	0.887647	0.267285	0.750639	0.037*	
C23	1.0639 (3)	0.39888 (15)	0.71301 (8)	0.0298 (4)	
H23A	1.146748	0.411157	0.676381	0.036*	
H23B	0.929314	0.453473	0.713353	0.036*	
C24	1.2429 (4)	0.41756 (16)	0.76706 (8)	0.0312 (4)	
H24A	1.356372	0.353377	0.771095	0.037*	
H24B	1.152369	0.419553	0.803024	0.037*	
C25	1.3899 (4)	0.52512 (16)	0.76421 (9)	0.0335 (4)	
H25A	1.478246	0.524128	0.727887	0.040*	
H25B	1.277315	0.589698	0.761315	0.040*	
C26	1.5718 (4)	0.54024 (17)	0.81829 (9)	0.0356 (4)	
H26A	1.677894	0.473452	0.822144	0.043*	
H26B	1.481523	0.544120	0.854257	0.043*	
C27	1.7314 (4)	0.64393 (17)	0.81632 (9)	0.0374 (4)	
H27A	1.819955	0.641269	0.780050	0.045*	
H27B	1.626618	0.711228	0.813756	0.045*	
C28	1.9139 (4)	0.65407 (19)	0.87038 (10)	0.0439 (5)	
H28A	1.824831	0.657064	0.906555	0.053*	
H28B	2.017259	0.586294	0.873072	0.053*	
C29	2.0762 (4)	0.7564 (2)	0.86910 (11)	0.0512 (6)	
H29A	2.192124	0.756753	0.904500	0.077*	
H29B	1.976150	0.824116	0.868607	0.077*	
H29C	2.164945	0.754289	0.833362	0.077*	

### Atomic displacement parameters (Å^2^)

**Table:**

	*U*^11^	*U*^22^	*U*^33^	*U*^12^	*U*^13^	*U*^23^
O1	0.0295 (7)	0.0286 (6)	0.0409 (8)	0.0049 (5)	0.0046 (6)	−0.0024 (6)
N1	0.0274 (7)	0.0221 (7)	0.0306 (8)	0.0014 (6)	0.0041 (6)	0.0023 (6)
N2	0.0252 (7)	0.0233 (7)	0.0284 (7)	−0.0016 (6)	0.0024 (6)	0.0016 (6)
N3	0.0276 (7)	0.0312 (8)	0.0334 (8)	0.0053 (7)	0.0012 (6)	0.0029 (7)
N4	0.0281 (8)	0.0429 (9)	0.0365 (9)	0.0031 (7)	0.0058 (7)	0.0046 (8)
N5	0.0268 (7)	0.0394 (9)	0.0372 (9)	0.0005 (7)	0.0053 (7)	0.0046 (7)
N6	0.0252 (7)	0.0291 (7)	0.0283 (7)	−0.0042 (6)	0.0013 (6)	−0.0016 (6)
N7	0.0272 (8)	0.0605 (11)	0.0331 (8)	−0.0084 (8)	0.0041 (7)	−0.0057 (8)
N8	0.0254 (8)	0.0553 (11)	0.0326 (8)	−0.0058 (7)	0.0043 (6)	−0.0065 (8)
C1	0.0250 (8)	0.0263 (8)	0.0263 (9)	0.0005 (7)	−0.0020 (7)	−0.0012 (7)
C2	0.0370 (10)	0.0240 (8)	0.0325 (10)	−0.0010 (8)	−0.0052 (8)	−0.0006 (7)
C3	0.0426 (11)	0.0261 (9)	0.0370 (11)	0.0072 (8)	−0.0061 (9)	−0.0065 (8)
C4	0.0349 (10)	0.0392 (11)	0.0339 (10)	0.0089 (8)	−0.0013 (8)	−0.0104 (8)
C5	0.0301 (9)	0.0338 (10)	0.0283 (9)	0.0017 (7)	0.0019 (7)	−0.0022 (7)
C6	0.0249 (8)	0.0248 (8)	0.0264 (8)	0.0015 (7)	−0.0024 (7)	−0.0015 (7)
C7	0.0234 (8)	0.0256 (8)	0.0271 (8)	−0.0011 (7)	−0.0011 (7)	−0.0019 (7)
C8	0.0283 (8)	0.0243 (8)	0.0327 (9)	−0.0028 (7)	−0.0002 (7)	0.0021 (7)
C9	0.0230 (8)	0.0234 (7)	0.0344 (9)	0.0002 (7)	0.0038 (7)	0.0051 (7)
C10	0.0250 (8)	0.0331 (9)	0.0335 (9)	0.0020 (8)	0.0043 (7)	0.0038 (8)
C11	0.0380 (10)	0.0366 (10)	0.0332 (10)	0.0079 (9)	−0.0042 (8)	−0.0013 (8)
C12	0.0292 (9)	0.0325 (9)	0.0326 (9)	0.0031 (7)	0.0005 (8)	0.0033 (7)
C13	0.0330 (10)	0.0329 (10)	0.0312 (9)	0.0038 (7)	0.0002 (8)	−0.0001 (7)
C14	0.0315 (9)	0.0312 (9)	0.0341 (10)	0.0027 (8)	−0.0015 (8)	−0.0008 (7)
C15	0.0346 (10)	0.0339 (10)	0.0326 (10)	0.0034 (8)	−0.0019 (8)	−0.0010 (7)
C16	0.0337 (10)	0.0334 (9)	0.0359 (10)	0.0026 (8)	−0.0018 (8)	0.0016 (8)
C17	0.0437 (12)	0.0425 (12)	0.0377 (11)	0.0081 (9)	−0.0052 (9)	0.0032 (9)
C18	0.0457 (12)	0.0443 (12)	0.0452 (13)	0.0109 (10)	−0.0005 (10)	0.0095 (10)
C19	0.0255 (8)	0.0317 (9)	0.0291 (9)	−0.0070 (7)	0.0012 (7)	0.0016 (7)
C20	0.0244 (8)	0.0231 (8)	0.0299 (9)	−0.0015 (6)	0.0033 (7)	−0.0001 (6)
C21	0.0236 (8)	0.0261 (8)	0.0283 (8)	−0.0012 (7)	0.0034 (7)	0.0004 (7)
C22	0.0321 (9)	0.0331 (9)	0.0272 (9)	−0.0068 (8)	−0.0046 (7)	0.0003 (7)
C23	0.0301 (9)	0.0294 (9)	0.0291 (9)	−0.0031 (7)	−0.0019 (7)	−0.0011 (7)
C24	0.0326 (10)	0.0310 (9)	0.0295 (9)	−0.0050 (7)	−0.0010 (8)	−0.0016 (7)
C25	0.0317 (10)	0.0313 (9)	0.0367 (11)	−0.0065 (8)	−0.0014 (8)	−0.0010 (8)
C26	0.0342 (10)	0.0336 (10)	0.0381 (11)	−0.0068 (8)	−0.0023 (9)	−0.0014 (8)
C27	0.0338 (10)	0.0333 (10)	0.0443 (11)	−0.0068 (8)	−0.0022 (9)	−0.0027 (9)
C28	0.0423 (12)	0.0397 (11)	0.0480 (12)	−0.0102 (10)	−0.0053 (10)	−0.0034 (9)
C29	0.0436 (12)	0.0427 (12)	0.0649 (15)	−0.0125 (10)	−0.0094 (11)	−0.0077 (11)

### Geometric parameters (Å, º)

**Table:**

O1—C7	1.227 (2)	C14—H14B	0.9900
N1—C7	1.382 (2)	C15—C16	1.520 (3)
N1—C6	1.399 (2)	C15—H15A	0.9900
N1—C8	1.457 (2)	C15—H15B	0.9900
N2—C7	1.381 (2)	C16—C17	1.524 (3)
N2—C1	1.391 (2)	C16—H16A	0.9900
N2—C19	1.448 (2)	C16—H16B	0.9900
N3—N4	1.342 (2)	C17—C18	1.515 (3)
N3—C10	1.344 (2)	C17—H17A	0.9900
N3—C11	1.459 (2)	C17—H17B	0.9900
N4—N5	1.317 (2)	C18—H18A	0.9800
N5—C9	1.356 (2)	C18—H18B	0.9800
N6—N7	1.337 (2)	C18—H18C	0.9800
N6—C21	1.346 (2)	C19—C20	1.498 (2)
N6—C22	1.463 (2)	C19—H19A	0.9900
N7—N8	1.319 (2)	C19—H19B	0.9900
N8—C20	1.351 (2)	C20—C21	1.369 (2)
C1—C2	1.387 (2)	C21—H21	0.9500
C1—C6	1.399 (2)	C22—C23	1.523 (3)
C2—C3	1.392 (3)	C22—H22A	0.9900
C2—H2	0.9500	C22—H22B	0.9900
C3—C4	1.385 (3)	C23—C24	1.519 (2)
C3—H3	0.9500	C23—H23A	0.9900
C4—C5	1.399 (3)	C23—H23B	0.9900
C4—H4	0.9500	C24—C25	1.525 (3)
C5—C6	1.377 (3)	C24—H24A	0.9900
C5—H5	0.9500	C24—H24B	0.9900
C8—C9	1.496 (3)	C25—C26	1.523 (3)
C8—H8A	0.9900	C25—H25A	0.9900
C8—H8B	0.9900	C25—H25B	0.9900
C9—C10	1.373 (2)	C26—C27	1.524 (3)
C10—H10	0.9500	C26—H26A	0.9900
C11—C12	1.512 (3)	C26—H26B	0.9900
C11—H11A	0.9900	C27—C28	1.519 (3)
C11—H11B	0.9900	C27—H27A	0.9900
C12—C13	1.524 (3)	C27—H27B	0.9900
C12—H12A	0.9900	C28—C29	1.518 (3)
C12—H12B	0.9900	C28—H28A	0.9900
C13—C14	1.522 (3)	C28—H28B	0.9900
C13—H13A	0.9900	C29—H29A	0.9800
C13—H13B	0.9900	C29—H29B	0.9800
C14—C15	1.522 (2)	C29—H29C	0.9800
C14—H14A	0.9900		
			
C7—N1—C6	109.97 (14)	C15—C16—C17	114.31 (17)
C7—N1—C8	123.91 (14)	C15—C16—H16A	108.7
C6—N1—C8	126.02 (14)	C17—C16—H16A	108.7
C7—N2—C1	110.01 (14)	C15—C16—H16B	108.7
C7—N2—C19	122.99 (15)	C17—C16—H16B	108.7
C1—N2—C19	126.99 (15)	H16A—C16—H16B	107.6
N4—N3—C10	111.05 (15)	C18—C17—C16	113.09 (17)
N4—N3—C11	120.59 (15)	C18—C17—H17A	109.0
C10—N3—C11	128.28 (15)	C16—C17—H17A	109.0
N5—N4—N3	106.89 (14)	C18—C17—H17B	109.0
N4—N5—C9	109.21 (15)	C16—C17—H17B	109.0
N7—N6—C21	110.88 (14)	H17A—C17—H17B	107.8
N7—N6—C22	119.89 (14)	C17—C18—H18A	109.5
C21—N6—C22	129.19 (15)	C17—C18—H18B	109.5
N8—N7—N6	107.11 (15)	H18A—C18—H18B	109.5
N7—N8—C20	108.94 (15)	C17—C18—H18C	109.5
C2—C1—N2	131.37 (18)	H18A—C18—H18C	109.5
C2—C1—C6	121.48 (17)	H18B—C18—H18C	109.5
N2—C1—C6	107.15 (15)	N2—C19—C20	112.93 (14)
C1—C2—C3	116.63 (18)	N2—C19—H19A	109.0
C1—C2—H2	121.7	C20—C19—H19A	109.0
C3—C2—H2	121.7	N2—C19—H19B	109.0
C4—C3—C2	121.84 (17)	C20—C19—H19B	109.0
C4—C3—H3	119.1	H19A—C19—H19B	107.8
C2—C3—H3	119.1	N8—C20—C21	108.28 (16)
C3—C4—C5	121.48 (18)	N8—C20—C19	120.19 (15)
C3—C4—H4	119.3	C21—C20—C19	131.51 (16)
C5—C4—H4	119.3	N6—C21—C20	104.79 (15)
C6—C5—C4	116.72 (18)	N6—C21—H21	127.6
C6—C5—H5	121.6	C20—C21—H21	127.6
C4—C5—H5	121.6	N6—C22—C23	112.32 (14)
C5—C6—N1	131.52 (16)	N6—C22—H22A	109.1
C5—C6—C1	121.85 (16)	C23—C22—H22A	109.1
N1—C6—C1	106.63 (15)	N6—C22—H22B	109.1
O1—C7—N2	126.84 (16)	C23—C22—H22B	109.1
O1—C7—N1	126.95 (16)	H22A—C22—H22B	107.9
N2—C7—N1	106.20 (14)	C24—C23—C22	110.72 (15)
N1—C8—C9	112.95 (15)	C24—C23—H23A	109.5
N1—C8—H8A	109.0	C22—C23—H23A	109.5
C9—C8—H8A	109.0	C24—C23—H23B	109.5
N1—C8—H8B	109.0	C22—C23—H23B	109.5
C9—C8—H8B	109.0	H23A—C23—H23B	108.1
H8A—C8—H8B	107.8	C23—C24—C25	113.69 (15)
N5—C9—C10	108.03 (16)	C23—C24—H24A	108.8
N5—C9—C8	121.92 (16)	C25—C24—H24A	108.8
C10—C9—C8	130.05 (16)	C23—C24—H24B	108.8
N3—C10—C9	104.82 (15)	C25—C24—H24B	108.8
N3—C10—H10	127.6	H24A—C24—H24B	107.7
C9—C10—H10	127.6	C26—C25—C24	112.51 (16)
N3—C11—C12	112.22 (15)	C26—C25—H25A	109.1
N3—C11—H11A	109.2	C24—C25—H25A	109.1
C12—C11—H11A	109.2	C26—C25—H25B	109.1
N3—C11—H11B	109.2	C24—C25—H25B	109.1
C12—C11—H11B	109.2	H25A—C25—H25B	107.8
H11A—C11—H11B	107.9	C25—C26—C27	114.60 (17)
C11—C12—C13	112.66 (15)	C25—C26—H26A	108.6
C11—C12—H12A	109.1	C27—C26—H26A	108.6
C13—C12—H12A	109.1	C25—C26—H26B	108.6
C11—C12—H12B	109.1	C27—C26—H26B	108.6
C13—C12—H12B	109.1	H26A—C26—H26B	107.6
H12A—C12—H12B	107.8	C28—C27—C26	112.76 (17)
C14—C13—C12	112.57 (15)	C28—C27—H27A	109.0
C14—C13—H13A	109.1	C26—C27—H27A	109.0
C12—C13—H13A	109.1	C28—C27—H27B	109.0
C14—C13—H13B	109.1	C26—C27—H27B	109.0
C12—C13—H13B	109.1	H27A—C27—H27B	107.8
H13A—C13—H13B	107.8	C29—C28—C27	113.62 (19)
C13—C14—C15	114.46 (15)	C29—C28—H28A	108.8
C13—C14—H14A	108.6	C27—C28—H28A	108.8
C15—C14—H14A	108.6	C29—C28—H28B	108.8
C13—C14—H14B	108.6	C27—C28—H28B	108.8
C15—C14—H14B	108.6	H28A—C28—H28B	107.7
H14A—C14—H14B	107.6	C28—C29—H29A	109.5
C16—C15—C14	113.30 (16)	C28—C29—H29B	109.5
C16—C15—H15A	108.9	H29A—C29—H29B	109.5
C14—C15—H15A	108.9	C28—C29—H29C	109.5
C16—C15—H15B	108.9	H29A—C29—H29C	109.5
C14—C15—H15B	108.9	H29B—C29—H29C	109.5
H15A—C15—H15B	107.7		
			
C10—N3—N4—N5	0.6 (2)	N4—N5—C9—C10	0.3 (2)
C11—N3—N4—N5	177.67 (15)	N4—N5—C9—C8	−179.02 (16)
N3—N4—N5—C9	−0.6 (2)	N1—C8—C9—N5	−113.96 (19)
C21—N6—N7—N8	−0.7 (2)	N1—C8—C9—C10	66.8 (2)
C22—N6—N7—N8	177.02 (16)	N4—N3—C10—C9	−0.4 (2)
N6—N7—N8—C20	0.2 (2)	C11—N3—C10—C9	−177.17 (17)
C7—N2—C1—C2	−178.80 (18)	N5—C9—C10—N3	0.0 (2)
C19—N2—C1—C2	0.0 (3)	C8—C9—C10—N3	179.31 (18)
C7—N2—C1—C6	1.31 (19)	N4—N3—C11—C12	−107.0 (2)
C19—N2—C1—C6	−179.84 (15)	C10—N3—C11—C12	69.6 (2)
N2—C1—C2—C3	−179.77 (18)	N3—C11—C12—C13	−177.95 (16)
C6—C1—C2—C3	0.1 (3)	C11—C12—C13—C14	169.67 (17)
C1—C2—C3—C4	0.0 (3)	C12—C13—C14—C15	178.35 (16)
C2—C3—C4—C5	−0.5 (3)	C13—C14—C15—C16	175.90 (17)
C3—C4—C5—C6	0.9 (3)	C14—C15—C16—C17	−179.27 (17)
C4—C5—C6—N1	179.66 (17)	C15—C16—C17—C18	176.78 (18)
C4—C5—C6—C1	−0.7 (3)	C7—N2—C19—C20	−88.9 (2)
C7—N1—C6—C5	178.61 (18)	C1—N2—C19—C20	92.4 (2)
C8—N1—C6—C5	−4.8 (3)	N7—N8—C20—C21	0.3 (2)
C7—N1—C6—C1	−1.08 (18)	N7—N8—C20—C19	178.80 (16)
C8—N1—C6—C1	175.52 (16)	N2—C19—C20—N8	159.08 (17)
C2—C1—C6—C5	0.2 (3)	N2—C19—C20—C21	−22.8 (3)
N2—C1—C6—C5	−179.86 (15)	N7—N6—C21—C20	0.8 (2)
C2—C1—C6—N1	179.96 (16)	C22—N6—C21—C20	−176.61 (16)
N2—C1—C6—N1	−0.14 (18)	N8—C20—C21—N6	−0.6 (2)
C1—N2—C7—O1	177.12 (17)	C19—C20—C21—N6	−178.96 (17)
C19—N2—C7—O1	−1.8 (3)	N7—N6—C22—C23	−106.9 (2)
C1—N2—C7—N1	−1.95 (18)	C21—N6—C22—C23	70.3 (2)
C19—N2—C7—N1	179.15 (14)	N6—C22—C23—C24	177.04 (15)
C6—N1—C7—O1	−177.20 (17)	C22—C23—C24—C25	168.59 (16)
C8—N1—C7—O1	6.1 (3)	C23—C24—C25—C26	−178.67 (17)
C6—N1—C7—N2	1.86 (18)	C24—C25—C26—C27	177.38 (17)
C8—N1—C7—N2	−174.83 (15)	C25—C26—C27—C28	−178.60 (18)
C7—N1—C8—C9	−113.03 (18)	C26—C27—C28—C29	179.6 (2)
C6—N1—C8—C9	70.8 (2)		

### Hydrogen-bond geometry (Å, º)

*Cg*2 is the centroid of the N3–N5/C9/C10 ring.

**Table:**

*D*—H···*A*	*D*—H	H···*A*	*D*···*A*	*D*—H···*A*
C2—H2···O1^i^	0.95	2.59	3.502 (2)	162
C10—H10···N5^ii^	0.95	2.44	3.317 (2)	153
C19—H19*A*···O1^i^	0.99	2.43	3.334 (2)	152
C21—H21···N8^iii^	0.95	2.62	3.372 (2)	137
C22—H22*A*···*Cg*2^iv^	0.99	2.89	3.664 (2)	135

Symmetry codes: (i) −*x*+1, *y*−1/2, −*z*+1; (ii) *x*−1, *y*, *z*; (iii) *x*+1, *y*, *z*; (iv) −*x*+2, *y*−1/2, −*z*+1.

### Comparison of the selected (X-ray and DFT) geometric data (Å, °)

**Table:**

Bonds/angles	X-ray	B3LYP/6-311G(d,p)
O1—C7	1.227 (2)	1.2256
N1—C7	1.382 (2)	1.3862
N1—C6	1.399 (2)	1.3954
N1—C8	1.457 (2)	1.4529
N2—C7	1.381 (2)	1.3838
N2—C1	1.391 (2)	1.3957
N3—N4	1.342 (2)	1.3447
N4—N5	1.317 (2)	1.3118
N4—N3—C10	111.05 (15)	111.56
N4—N3—C11	120.59 (15)	120.32
C10—N3—C11	128.28 (15)	128.09
N5—N4—N3	106.89 (14)	106.96
N4—N5—C9	109.21 (15)	109.85

### Calculated energies.

**Table:**

Molecular Energy (a.u.) (eV)	Compound (I)
Total Energy *TE* (eV)	–44752.35
E_HOMO_ (eV)	–5.72
E_LUMO_ (eV)	–0.68
Gap *ΔE* (eV)	5.04
Dipole moment *µ* (Debye)	3.96
Ionisation potential *I* (eV)	5.72
Electron affinity *A*	0.68
Electronegativity *χ*	3.202
Hardness *η*	2.19
Softness *σ*	0.19
Electrophilicity index *ω*	2,03
