# Supplementary material for: Synthesis, structure and Hirshfeld surface analysis of 1,3-bis­[(1-octyl-1H-1,2,3-triazol-4-yl)meth­yl]-1H-benzo[d]imidazol-2(3H)-one
Source: Acta Crystallogr E Crystallogr Commun. 2023 Nov 21;79(Pt 12):1179–82. doi: 10.1107/S2056989023009891 (PMC10833399; doi:10.1107/S2056989023009891)
Supplement: Supplementary file 5 [file e-79-01179-sup4.docx]

Supplementary Figure S1: void volumes

Supplementary Figure S2: energy frameworks


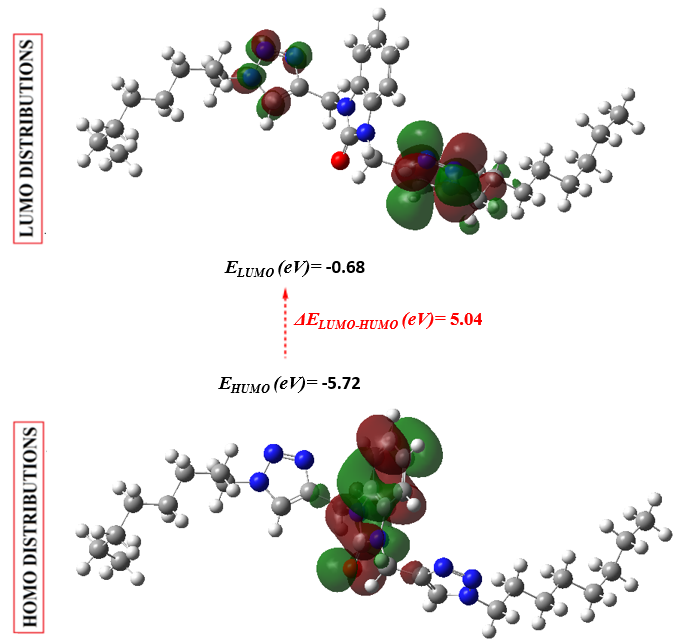


Supplementary Figure S3: HOMO and LUMO and energy gap.
